# Modulated-Diameter Zirconia Nanotubes for Controlled Drug Release—Bye to the Burst

**DOI:** 10.3390/jfb16020037

**Published:** 2025-01-21

**Authors:** Gabriel Onyenso, Swathi Naidu Vakamulla Raghu, Patrick Hartwich, Manuela Sonja Killian

**Affiliations:** Chemistry and Structure of Novel Materials, University of Siegen, Paul-Bonatz-Str. 9-11, 57076 Siegen, Germany; gabriel.onyenso@student.uni-siegen.de (G.O.); patrick.hartwich@uni-siegen.de (P.H.)

**Keywords:** oxide nanostructures, zirconia, controlled release drug delivery, anodization

## Abstract

The performance of an orthopedic procedure depends on several tandem functionalities. Such characteristics include materials’ surface properties and subsequent responses. Implant surfaces are typically roughened; this roughness can further be optimized to a specific morphology such as nanotubular roughness (ZrNTs) and the surfaces can further be used as static drug reservoirs. ZrNTs coatings are attracting interest due to their potential to improve the success rate of implant systems, by means of better physical affixation and also micro/nano physio-chemical interaction with the extracellular matrix (ECM). Effective control over the drug release properties from such coatings has been the subject of several published reports. In this study, a novel and simple approach to extending drug release time and limiting the undesirable burst release from zirconia nanotubes (ZrNTs) via structural modification was demonstrated. The latter involved fabricating a double-layered structure with a modulated diameter and was achieved by varying the voltage and time during electrochemical anodization. The structurally modified ZrNTs and their homogenous equivalents were characterized via SEM and ToF-SIMS, and their drug release properties were monitored and compared using UV–Vis spectroscopy. We report a significant reduction in the initial burst release phenomenon and enhanced overall release time. The simple structural modification of ZrNTs can successfully enhance drug release performance, allowing for flexibility in designing drug delivery coatings for specific implant challenges, and offering a new horizon for smart biomaterials based on metal oxide nanostructures.

## 1. Introduction

Since its conception in the mid-1960s, when it was shown that a silicone capsule with constant dimension had the potential to deliver a drug at a constant rate [1,2], the concept of localized controlled drug delivery now affects millions of patients across the world. This approach to drug administration has become favorable as it addresses some of the limitations of the conventional delivery methods, e.g., oral, injected, or subcutaneous, such as poor biodistribution, lack of selectivity, toxicity, issues relating to metabolism, and potentially, poor patient compliance, etc. [3]. An increasing need for orthopedic implant applications has driven demands for products focused on improving patient comfort and care. In light of this, recent investigations into the integration of drug-eluting implant surfaces have reportedly enhanced the fixation of implants [4]. The strategy of fabricating drug-eluting surfaces on fixed implants has seen an evolution from conventionally employed drug delivery methodologies using macroscopic delivery devices like mucosal inserts, bandages, and ingestible capsules, to subsequent progression in the 1990s when microscopic degradable polymers were commercialized, to the currently active trend of integrating nanotechnology into drug delivery [1,5,6,7].

A key area at the center of this integration is the use of nanoporous/nanotubular structures of metal oxides as vessels for controlled and targeted loading with and release of drugs, biomolecules, etc. [3]. Such nanostructured materials have attracted attention due to their chemical stability [8], good wettability [9], biocompatibility, mechanical robustness, and tendency to bio-mimic the ECM (extracellular matrix), promoting osseointegration, to name a few of their functionalities [10,11,12,13]. In addition to a large inner volume, nanotubular structures consist of distinct inner and outer surfaces that can be further modified with a range of drug molecules and proteins to elicit additional responsivity [14,15]. These structures have been reported to be transferrable to ceramic zirconia surfaces [16] and are particularly useful in orthopedic implant systems, where both structural and chemical coating of implants may effectively address problems of inflammation, poor osseointegration, and bacterial infection affecting implant systems [17,18,19].

Zirconia nanotubes (ZrNTs) are promising candidates for such delivery coatings, particularly on Zr or zirconia-based implants. The concepts relating to their fabrication are closely related to other extensively studied nanostructured materials like nanoporous anodic alumina (NAA) and titania nanotubes (TiNTs) [3,10,20]. Zirconia was introduced as a biomaterial along with other classes of high-performance ceramics, as an alternative to address the drawbacks of alumina in orthopedic applications [21,22,23], due to its chemical inertness, biocompatibility, and mechanical strength [24,25]. Particularly in dental implants, zirconia is considered more suitable due to favorable properties such as white coloration, translucency, and generally good aesthetics [21,26,27]. A highly ordered array of ZrNTs can be fabricated by electrochemical anodization of zirconium in a fluoride-containing electrolyte, yielding defined dimensions and geometry [13,28,29,30]. Previous work on drug release from nanotubes has concluded that the diffusion process depends significantly on the dimensions of the nanotubes, in addition to factors like the molecular size and charge of the drug, drug solubility, pH, etc. [31,32,33,34,35]. The release kinetics from these structures consist mostly of an initial burst release followed by a constant gradual zero-order kinetic mechanism [36,37,38]. This initial burst release is undesirable because the amount released in most cases is higher than the maximum effective level and can be toxic to the surrounding body tissues. Various strategies such as structural modification of the nanotubes, reducing pore size via polymer deposition, surface functionalization, etc., have been explored to limit the burst effect and extend the release time in zero-order release kinetics [37,39]. General outcomes from previous findings have revealed that an increase in tube dimensions (i.e., thickness) translates to extended release time. However, increasing other morphological dimensions, such as the pore diameter, also increases the initial burst release, while a smaller constant pore diameter with similar thickness minimizes the loading and release capacity [3,40]. Attempts at reducing the diameter of the pore openings via biopolymer deposition have also been investigated. While this approach may have significantly reduced the initial burst release and extended the release time [41,42,43], it is suspected that the deposited polymers may potentially degrade over time and release their monomer in the body, leading to possible inflammation and subsequent implant failure [44,45]. Furthermore, coating with a polymer may change the surface roughness and chemistry at the cell–implant interacting surface, potentially improving interaction with the ECM due to suppression of the underlying nanostructural morphology, which has been proven to demonstrate enhanced biocompatibility [46,47]. In this work, we attempted to optimize the loading capacity and subsequent release capabilities via structural modification, without comprising direct exposure to the desired nanostructural morphology.

**Scheme 1:**
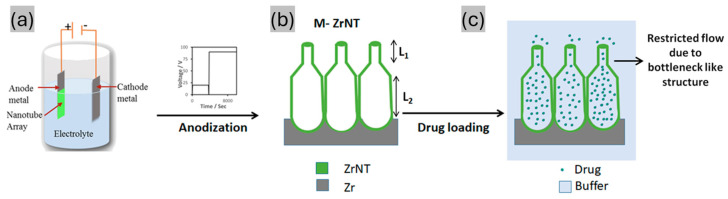
Scheme depicting the methodology for fabricating double-layered ZrNTs with varying diameters for enhanced drug release performance: (**a**) electrochemical anodization in two-step voltage conditions; (**b**) double-layered ZrNTs with varying diameters; (**c**) drug release from these modified structures.

This was achieved by the electrochemical fabrication of a double-layer (bottleneck) structure (Scheme 1) wherein the first layer had the desired reduced pore diameter to control the initial burst release and encourage cellular integration, opening into a second layer with a much larger diameter that functioned as the primary storage vessel. This second layer was found to be capable of accommodating a large quantity of drug molecules, hence improving the loading capacity and in general, influencing the release performance. The double-layer modulated thickness was fabricated based on the previous reported works on TiNT and NAA. Stepwise alternation of the anodic voltage and the consequent current density was shown to disrupt the immediate electrochemical equilibrium, resulting in a change in morphology within the anodic structure. This phenomenon, previously observed for TiNT and NAA [48,49,50,51,52], was for the first time reciprocated in ZrNTs to create double-layered structures with different diameters. The drug release performance from these double-layered structures was compared with structures that included constant tubular length and pore diameter. Suppressed burst release and, generally, prolonged release could be achieved.

## 2. Materials and Methods

### 2.1. Fabrication of Zirconia Nanotubes (ZrNTs)

Zirconium foil (99.5% purity, 0.127 mm thickness) was ultrasonicated in acetone, ethanol, and de-ionized water for 10 min each and dried in N_2_ prior to electrochemical anodization. The anodization comprised a one-pot synthesis using a high-voltage potentiostat (Jaissle IMP 88—200 PC, IPS-Elektronik Labor, Muenster, Germany). An electrochemical cell was used, consisting of a circular working area of 1 cm^2^ and a platinum counter electrode in a typical two-electrode setup. The organic electrolyte was based on the previous work by Vakamulla et al. and contained 2 wt% NH_4_F (Sigma Aldrich, Merck KGaA, Schnelldorf, Germany), 2 wt% H_2_O, and 30% formamide (Carl Roth, Karlsruhe, Germany) in glycerol (Carl Roth, Karlsruhe, Germany) [9]. Homogenously thick ZrNTs were fabricated by ramping the potential from 0 to 90 V over 60 s, followed by holding at 90 V for 2 h. The two types of modulated ZrNT structures (M_1_ and M_2_) were anodized under two voltage regimes (20–90 V). Preparation of the type 1 modulated structure (M_1_) involved ramping the voltage to 20 V for 60 s and holding at 20 V for 1 h to obtain the first nanotubular layer. Thereafter, the bias was ramped down to 0 V for 60 s and subsequently, the voltage was ramped up again to 90 V for 60 s and held for 2 h to achieve the second layer. Preparation of the type 2 modulated structure (M_2_) entailed the same voltage conditions as M_1_ to obtain the second layer, but the first layer was obtained by anodizing at 20 V for 4 h, after ramping up gradually for 60 s. The current density and voltage during anodization were recorded and monitored using ECM-Win software, EcmWin. Post-anodization, the samples were rinsed with deionized water and soaked in ethanol for 10 min to dissolve the remains from the organic electrolyte, followed by annealing at 450 °C for 1 h, aiming towards the elimination of fluoride present in the as-formed nanotubes whilst improving the phase crystallinity of the NTs [53]. X-ray diffraction measurements (XRD; X’Pert Pro, PANalytical, Malvern, Kassel, Germany) with Cu Kα radiation) were carried out to further characterize the samples.

### 2.2. Drug Loading and Release

All ZrNTs were cleaned by washing with ethanol and deionized water and were dried with N_2_ prior to drug loading. Diclofenac sodium (DCF) was selected as a model drug, and loading was carried out by pipetting 50 µL of a 0.045 mM solution of the drug in ethanol onto the active surface area of the nanotubes (1 cm^2^). After pipetting, the ZrNTs were left undisturbed for 1.5 min, enabling diffusion-dependent deposition into the tubes, followed by gentle heating at ~50 °C to evaporate the solvent. This step was repeated 20 times to load a substantial quantity of the drug molecules. The last step in the loading process involved rinsing the ZrNT surface with deionized water and drying it in N_2_.

The study of drug release was conducted by measuring the absorbance of the released drug at the characteristic maximum wavelength of DCF (224 nm) using a UV–Vis spectrophotometer (Lambda XLS/XLS + Perkin Elmer, Becaonsfield, UK). The drug-loaded samples were immersed in a pure ethanol solvent (10 mL). At specific intervals, 2 mL samples of the solution were pipetted out for absorbance measurement, and fresh ethanol was transferred back to the release medium to maintain a constant volume. Absorbance measurements were carried out every 10 min for the first 4 h, followed by measurement every 24 h until there were no detectable changes in the absorbance. The amount of drug loaded was calculated from the absorbance values using the calibration curve for DCF in ethanol. The loading capacity was calculated by summing the amounts of the drug released at various time points until no drug was released.

### 2.3. Chemical Characterization of Drug-Loaded ZrNTs

For analyzing the depth distribution of DCF within the nanotubes, a combined method including focused ion beam (FIB) sputtering and time-of-flight secondary ion mass spectrometry (ToF-SIMS) was used. An inclined plane was milled into the filled nanotubes via FIB (DualBeam Helios NanoLab 600, FEI, Thermo Fischer, Dreieich, Germany), ranging from the top surface to the metallic Zr substrate across an area of 50 × 100 mm^2^. The beam parameters were 16 kV acceleration voltage and 21 nA beam current. The milling was performed in three consecutive steps, each successively increasing the depth by reducing the milled area. (The schematic for the ion milling is shown in the Supporting Information, Figure S1). This was necessary to reduce the heating and fragmentation of the organic constituents of the sample. The crater, created at an incline, was analyzed in the negative polarity according to characteristic secondary ion mass fragmentation (e.g., F^−^ (*m*/*z* 19.00), CN^−^ (*m*/*z* 26.01), CNO^−^ (*m*/*z* 41.99), Cl^−^ (*m*/*z* 34.97), CHO_2_^−^ (*m*/*z* 45.02)), with respect to ZrNTs and DCF. Chemical mapping images were recorded with ToF-SIMS (TOF–SIMS 4, IONTOF GmbH, Muenster, Germany). For imaging, Bi_3_^+^ ions with an accelerated voltage of 25,000 eV were used. The sample stage was tilted at 4 ° towards the primary ion beam to avoid shadowing effects and to increase the impact angle of the probe. Principal component analysis was conducted with Spectragui software (NESAC/BIO, UW Seattle, Seattle, WA, USA); data were normalized and root-mean-square centered.

## 3. Results

### 3.1. Fabrication and Characterization of ZrNT

**Figure 1:**
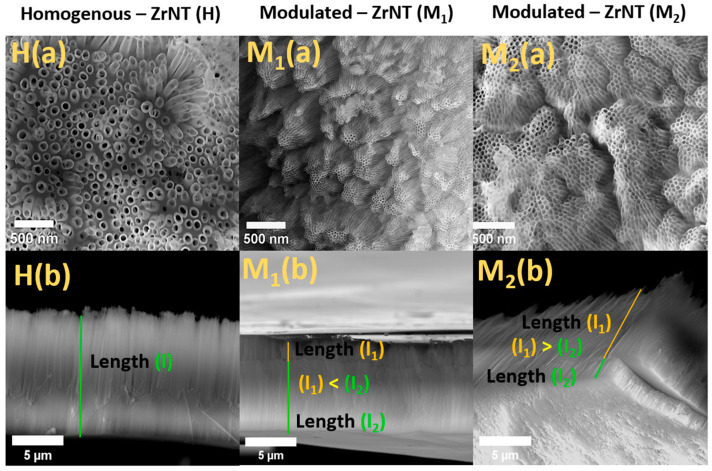
SEM micrographs: Row 1, top view of ZrNTs (homogenous diameter, H-ZrNTs; modulated type 1, M_1_-ZrNTs; modulated type 2, M_2_-ZrNTs; Row 2, cross-sectional view of ZrNTs (H-ZrNTs—uniform length (l); M_1_-ZrNTs—l_1_ < l_2_; M_2_-ZrNTs—l_1_ > l_2_).

**Figure 2:**
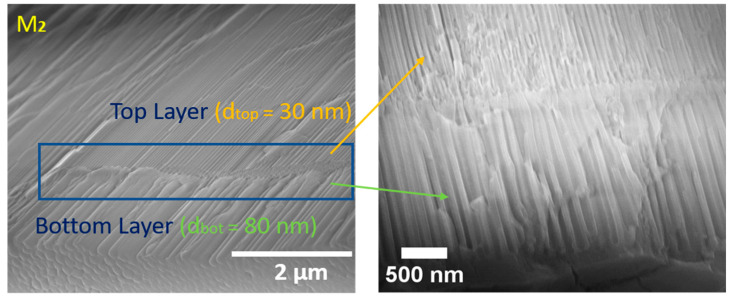
SEM micrographs: (**left**) cross-sectional view of M_2_; (**right**) high-magnification view of the interface.

**Figure 3:**
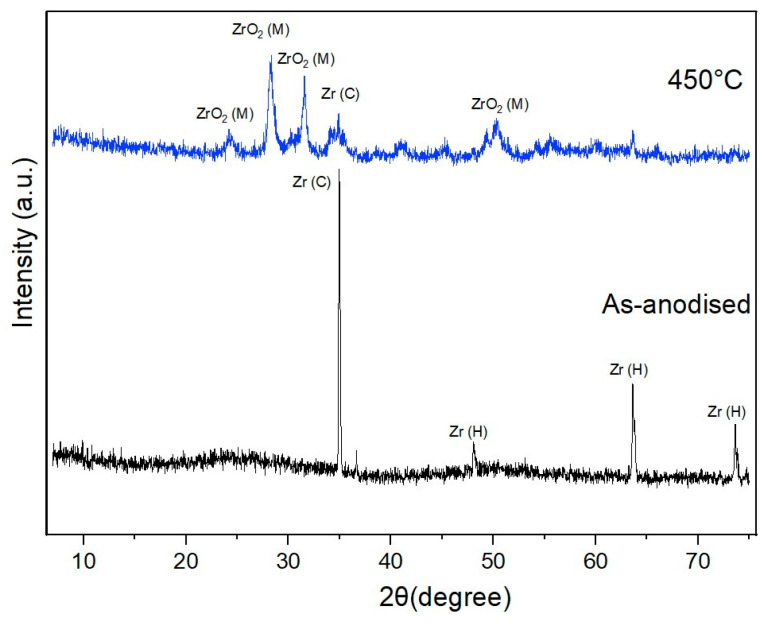
XRD spectra of ZrNTs as anodized and annealed at 450 °C.

ZrNTs were fabricated and structurally characterized using SEM. Images showing their top and cross-sectional views are summarized in Figure 1. Two sets of ZrNTs were fabricated: homogenous and modulated with tight openings. The SEM images from the top view indicate tightly packed open tubular structures with average diameters of 80 ± 20 nm and 30 ± 5 nm for the homogenous and diameter-modulated (bottleneck) structures, respectively. The cross-sectional view depicts an approximate thickness of 9 µm for both structures. The homogenous structure includes a smooth thickness across the length with no distinct variation in diameter along the tube. Conversely, for the modulated structure, a double layer can be observed, with different diameters for each layer (top layer d_1_, ~30 ± 10 nm and bottom layer d_2_, 80 ± 20 nm), as a result of varying the applied bias during the anodization. Two different bottle-neck structures were produced, showing variation in lengths. For M_1_, dimensions of l_1_ ≤ 2 μm and l_2_ ≤ 7 μm and for M_2_, thicknesses of l_1_ ≤ 7 μm and l_2_ ≤ 2 μm were achieved as a result of tuning the anodization time at the respective voltages. An SEM image of the modulated interface is represented in Figure 2 and the variation in morphology is summarized in Table 1. The ZrNTs were also characterized with XRD to determine the crystallinity of the zirconia layer; Figure 3 compares the patterns of the annealed and non-annealed ZrNTs. The diffraction peaks at 2θ = 35°, 48°, 63°, 78° observed in the non-annealed samples are from the zirconium (Zr) substrate, indexed as the cubic and hexagonal phase of Zr [54,55], indicating the amorphous structure of the anodized ZrNTs. The annealed sample contained peaks mainly from the monoclinic zirconia crystalline phase at 24°, 28°, 31°, and 50° [56]. This confirmed that annealing up to 450 °C led to the transformation of the ZrNTs from an amorphous to a crystalline structure.

### 3.2. Drug Loading and Release Characteristics

The three sets of fabricated ZrNTs were subjected to drug loading and release studies. Diclofenac sodium (DCF), a nonsteroidal anti-inflammatory drug, was used in these experiments [57]. DCF was selected mainly due to its cost efficiency and pharmaceutical efficacy and availability. It is a well-established medication for treating inflammation and pain, especially in orthopaedic applications. The current work aimed to demonstrate that the release properties from ZrNTs could be enhanced by modifying the homogenous length to a bottleneck-like structure. Different drugs can be used depending on the intended purpose, for applications such as anticancer, anti-inflammatory, anti-fungal treatment, etc., including multi-drug systems. In this work, DCF was used as a model drug to demonstrate one such possibility, with the ZrNTs as the platform for the drug delivery. The structures are denoted as follows: homogeneously thick unmodulated ZrNTs (9 µm tube length and 80 nm ± 20 nm diameter) are identified as H-ZrNTs, while the two variations of the modulated bottle-neck structures are referred to as M_1_-ZrNTs (l_1_ = 2 µm, 30 nm ± 5 nm diameter, l_2_ = 7 µm, 80 nm ± 20 nm diameter) and M_2_-ZrNTs (l_1_ = 7 µm, 30 nm ± 5 nm diameter; l_2_ = 2 µm, 80 nm ± 20 nm diameter).

**Figure 4:**
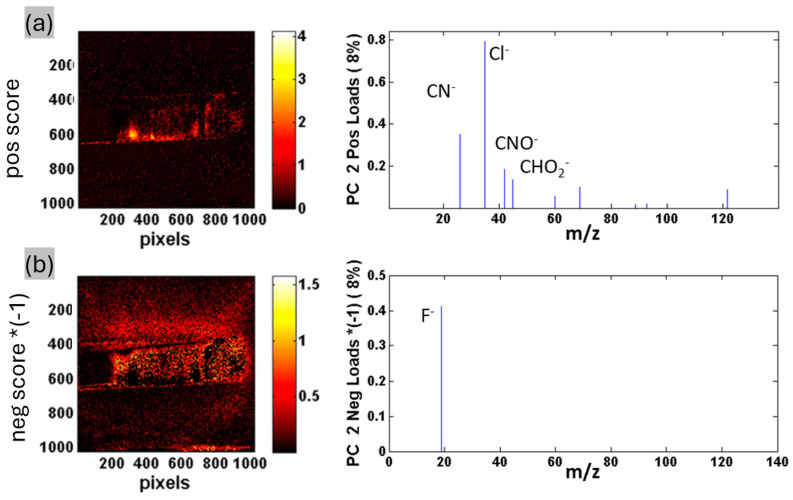
ToF-SIMS images of an inclined slope produced with a focused ion beam and subjected to principal component analysis. PC2 scores (positive (pos) and negative (neg *(-1)), and loadings showing (**a**) the characteristic DCF signals distributed along the entire ZrNT depth and (**b**) F^−^ originating from the ZrNTs (electrolyte residue).

To confirm that the drugs were loaded into the entire length of the tubes, chemical imaging along the depth of the nanotubes was conducted using a combined technique of focused ion beam (FIB) crater preparation and ToF-SIMS, a novel approach developed to reduce the depth profiling time, improve depth resolution, and avoid sputter artefacts in the analysis of hybrid inorganic–organic nanoarchitectures [58]. The FIB was used to create an inclined slope revealing the nanostructure from top to bottom (Figure S1a), allowing better resolution of the depth distribution of organics via ToF-SIMS imaging. The FIB crater included the whole nanostructure and part of the metal substrate, for convenient identification of the oxide–metal interface. The chemical distribution within the loaded nanotubes was analyzed using ToF-SIMS imaging of fragments characteristic of diclofenac, along the incline (Figure S1b,c). The intensely bright spots on the images may have been be caused by the various filling states of different sections of the ZrNTs, but are more likely to have been due to topographic artefacts [57,59]. To improve the interpretation of the ToF-SIMS images, in Figure 4, principal component analysis (PCA) was conducted. While the first principal component (PC1) contained solely topographic information (Figure S2), the PC2 separated the F^−^ signal indicative of the ZrNTs (residue from the electrolyte) from the characteristic DCF signals (CN^−^ (*m*/*z* 26.01), CNO^−^ (*m*/*z* 41.99), and Cl^−^ (*m*/*z* 34.97)). The characteristic fragments were present throughout the entire depth of the ZrNTs, confirming well-distributed DCF within the nanotubes.

## 4. Discussion

**Figure 5:**
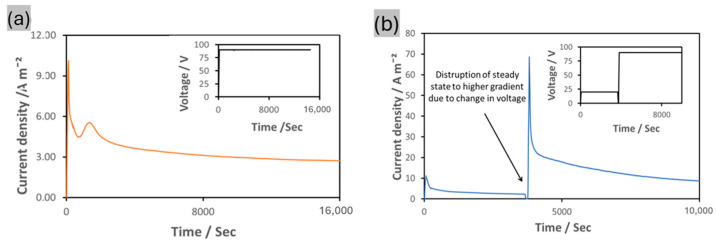
Current density–time curve for the anodization of Zr under conditions of (**a**) constant voltage and (**b**) modulated voltage. The inset shows the applied voltage–time conditions.

Electrochemical anodization of zirconium in a fluoride-containing electrolyte initially consists of the formation of a compact barrier oxide which undergoes dissolution at defect sites, mediated by the formation of a soluble zirconium fluoro-complex that enables porosification. These pores then serve as initiation spots, subsequently facilitating the growth of highly ordered porous anodic oxide. When an equilibrium state is established between the rate of oxidation and dissolution, stable nanotubular arrays are observed [51,60,61]. The anodization voltage is an important parameter that influences tube dimensions; as reported previously, the diameter increases with voltage and under a constant voltage regime, the diameter remains unchanged, as also shown in Figure 1. This concept of steady-state anodic growth at a constant potential resulting in a constant diameter was explored to fabricate double layers with different diameters. Varying the voltage resulted in perturbation of the localized electric field, causing a change in the dissolution kinetics. This subsequently created new pores and the increase in current density promoted pore growth, thereby resulting in the formation of larger diameters [62]. Therefore, by initiating nanotube formation at 20 V and thereafter increasing the voltage to 90 V, we were able to successfully fabricate double-layered ZrNTs with a modulated diameter. The thickness of both layers was controlled by altering the anodization time for each voltage regime. For the first layer, thicknesses of 2 µm and 7 µm were obtained by anodization for 1 h or 4 h, respectively. The current density curve provided electrochemical insight into the competing processes of oxidation and etching. The current density curve for the homogeneous ZrNTs showed an initial decrease (oxide layer formation) followed by an increase, indicating dissolution (initial pore formation) and subsequent passivation (Figure 5a). This result emphasizes the disruption of the steady-state condition due to the change to a higher voltage regime and the subsequent equilibration phase, adjusting to the new steady-state condition with a greater density of passivation current. These current density profiles are consistent with previously reported works on TiNTs, where periodic alternating voltage conditions were exploited to fabricate periodically modulated diameters for various applications [40,48,49,63]. Herein, we report for the first time the successful transposition of double-layered modulated anodic oxide structures onto a zirconium metal foil, generating a novel bottleneck structure.

The quantity of drug loaded in the H-ZrNTs was determined to be 4.96 ± 1.78 µg, which was less than the loading capacity observed for M_1_-ZrNTs (8.76 ± 0.63 µg) and M_2_-ZrNTs (10.22 ± 0.30 µg). The lower loading capacity observed in H-ZrNT might have been related to the increased interaction of the solvent with the drug molecules due to its larger diameter, leading to the elimination of more of the drug during the washing step in the loading process. Secondly, the effective packing density of nanotubes with a smaller tube diameter, as seen in Figure 1, would imply more ZrNTs per area available for covalent loading of the drug molecules [64]. Furthermore, as observed from the top view in the SEM images of the structures, the interpore distance was higher in the H-ZrNTs; this may possibly have translated to more drug molecules adhering at the surface rather than into the tubes and hence, easier washing off during the washing step.

**Figure 6:**
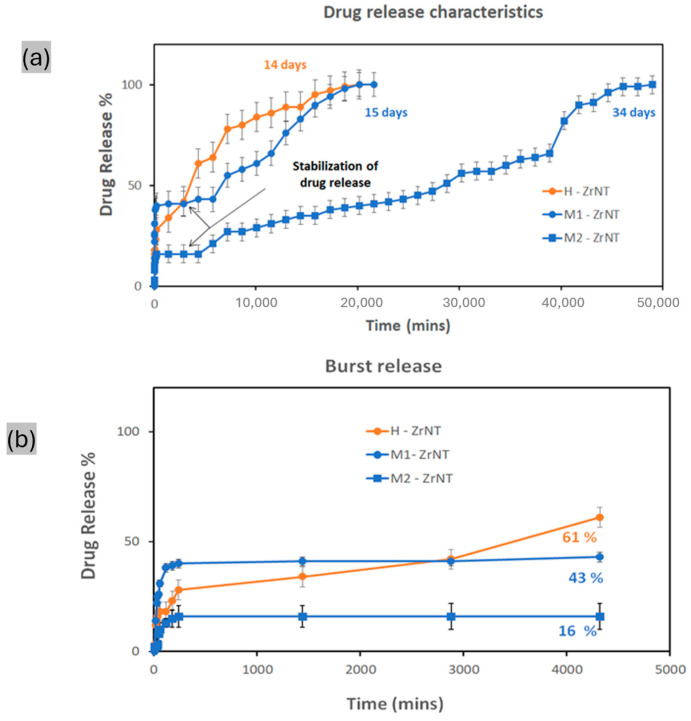
Drug release characteristics: (**a**) total drug release performance; (**b**) release during 3 days, visualizing the initial burst release characteristics.

The drug release from these structures was monitored and the cumulative release is graphically presented in Figure 6. Typically for such systems with concentration gradient, the release of the drug is mainly governed by diffusion of the molecules from the nanotubes (a region of high drug concentration) to the solvent medium (a region of low drug concentration) to establish a concentration equilibrium [3]. Hence, in the initial stages, a sudden release of these molecules occurs due to the higher concentration gradient. This phenomenon has a strong clinical implication, as for implants with a drug-eluting surface, sudden release may agitate the local environment and can result in an undesirable host–cell response. However, subsequently, the initial burst release gives way to more gradual zero-order release kinetics [65]. This typical, biphasic release behavior was also observed in the anodic nanostructures, as shown in Figure 6. However, comparing the release patterns from the three investigated platforms, differences in their behaviors were identified in terms of the initial burst release and the total release rate and time. The release characteristics revealed variation in the initial burst (i.e., the percentage released after 4 h); the initial release reduced from 28% to 16% for the H-ZrNTs and M_2_-ZrNTs, respectively. A possible explanation may be linked to the structural variation; in the H-ZrNTs with a higher pore size of 80 nm ± 20 nm, reduced restriction with a larger influx volume and immediate interaction with the solvent is expected, compared with the modulated structures with a pore diameter of 30 nm ± 20 nm at the top surface where, due to the smaller pore openings, more restricted diffusion is anticipated. These results are in good agreement with publications on structurally similar delivery systems, like TiNTs and NAA. Experiments reported by Losic et al. [41] showed that by reducing the pore size of the nanotube, the initial burst release was inhibited. However, as reported previously, the drawbacks of such an approach using unmodulated structures resulted in the lowering of the loading capacity as well. We addressed this limitation by introducing a double-layered system consisting of a top layer with reduced diameter. This layer minimizes the burst release and subsequently opens into a bottom layer with larger tube diameter for optimal storage of the drug. Additional loading into this layer is propelled by pressure differences within the adjoining capillaries. A summarized representation of these results is shown in Table 2. We achieved maximized loading capacity whilst lowering the burst release and subsequently prolonging the retention within the nanotubes via this double-layer modification, rather than by reducing the diameter along the entire length of the nanotubes. This is a viable solution to enable minimization of the burst release while not compromising on the loading efficiency. While the initial release from the M_1_-ZrNTs was higher (40%) compared with the H-ZrNTs, which may have been a consequence of the higher capillary forces caused by the smaller diameters, a close examination of the release curve showed stabilization of the drug release from the modulated structures after 4 h. Ultimately, the release from the H-ZrNTs was higher after 3 days, at 61%, compared with 43% for the M_1_-ZrNTs and 16% for the M_2_-ZrNTs. This stabilization may be attributed to an increased influence of the bottleneck between the first and second layers in the modulated structure in controlling the flow of drug. The time required for the drug to completely elute varied between the structures: 14 d for H-ZrNTs, 15 d for M_1_-ZrNTs, and a delayed time of 34 d for M_2_—ZrNTs. The differences in release time from the modulated structures (15 and 34 days for M_1_-ZrNTs and M_2_-ZrNTs, respectively) may have been caused by the length of the first layer, which increased from 2 µm to 7 µm, with the implication that it would take a much longer time for the drug loaded in the second layer to elute from the reservoir in the nanotubes, attributed to a longer diffusion path.

The release of therapeutics at a suitable dosage over a long period is a critical factor in integrating nanoporous or -tubular coatings in orthopedic applications. Various strategies have been investigated to achieve extended zero-order kinetic release of drugs. In this paper, as proof of concept, we have successfully shown that extended release of drugs can be achieved by simple structural modification of metal oxide nanostructures.

## 5. Conclusions

A simple strategy is described for enhancing the release performance from ZrNTs via structural modification. The modification involved the fabrication of a double-layered structure with a varying diameter, namely, a top layer with a smaller diameter opening into a second layer with a larger diameter. This bottle-neck structure was achieved by altering the voltage during anodization. Changing the voltage from 20 V to 90 V yielded a increase in diameter along the tube length from 30 nm ± 5 nm to 80 nm ± 20 nm. The results from the drug release studies indicated that the unwanted initial release was reduced by 74%, the total loading capacity was doubled, and the release time was extended by 143% when compared with an equivalent structure of constant diameter. This improved release behavior in terms of initial burst release and extended retention time indicates that the modulated double-layered structure with narrow openings and a large drug reservoir offers superior performance in its role as a drug delivery platform for implant systems. We have conclusively demonstrated that modulation of the diameter of ZrNTs can be obtained and that such modifications are suitable for drug delivery.

## Figures and Tables

**Table 1:** Summary of morphological parameters.

	Diameter (nm) (Top-Bottom)	Length (µm) (Top-Bottom)
H—ZrNT	80	9
M_1_—ZrNT	30 ± 5–80 ± 20	2–7
M_2_—ZrNT	30 ± 5–80 ± 20	7–2

**Table 2:** Drug release response—summary.

	H—ZrNT	M_1_—ZrNT	M_2_—ZrNT
Drug loading capacity (µg/cm^2^)	4.96	8.76	10.22
Loading efficency (%)	34.63	61.17	71.37
Initial burst release (wt%)	61	43	16
Total release time (days)	14	15	34

## Data Availability

The original contributions presented in the study are included in the article/Supplementary Material. Further inquiries can be directed to the corresponding authors.

## Supplementary Materials

The following supporting information can be downloaded at https://www.mdpi.com/article/10.3390/jfb16020037/s1. Figure S1: (a) back-scattered electron (BSE) image of the top view of the milled material created by FIB, indicating areas of nanostructure and metal substrate; (b,c) ToF-SIMS images showing distribution within the milled area of CHO_2_^−^ fragments (b) and Cl^−^ fragments (c) as indicators of the presence of DCF throughout the structure. The color scale corresponds to the fragments’ signal intensity. Figure S2: ToF-SIMS, PCA of inclined slope of FIB cut. PC1 contains solely topographic information (77% variance captured by PC1; (a) top surface, (b) all signals from FIB crater).
