# Supplementary material for: Modulated-Diameter Zirconia Nanotubes for Controlled Drug Release—Bye to the Burst
Source: J Funct Biomater. 2025 Jan 21;16(2):37. doi: 10.3390/jfb16020037 (PMC11856647; doi:10.3390/jfb16020037)
Supplement: Supplementary file 1 [file jfb-16-00037-s001.zip › jfb-3345602-supplementary.pdf]

Journal of
Functional Biomaterials

Supporting information
Modulated-Diameter Zirconia Nanotubes for Controlled Drug Release—Bye to the Burst

Gabriel Onyenso, Swathi Naidu Vakamulla Raghu *, Patrick Hartwich and Manuela Sonja Killian *

Chemistry and Structure of Novel Materials, University of Siegen, Paul-Bonatz-Str. 9-11, 57076 Siegen, Germany; gabriel.onyenso@student.uni-siegen.de (G.O.); patrick.hartwich@uni-siegen.de (P.H.)
* Correspondence: swathi.naidu@uni-siegen.de (S.N.V.R.); manuela.killian@uni-siegen.de (M.S.K.)

S1 Depth imaging of the ZrNT using a Combined FIB and ToF – SIMS

ToF – SIMS is a viable tool for analyzing the depth information of metal oxide nanotubes (e.g. ZrNT), however for very thick inorganic nanotubes (> 10 µm length), it has the limitation of increased analysis time and signal interference. FIB in combination with ToF – SIMS was introduced as an approach to limiting these drawbacks. This combination was adopted to obtain the chemical depth imaging along the ZrNT[58]. An inclined crater is produced on the nanostructure with a focused ion beam. The crater formed as shown from the top (Figure S1), serves to reduce the time required for image acquisition by ToF – SIMS and minimizes the signal interference along the depth of the nanotube.

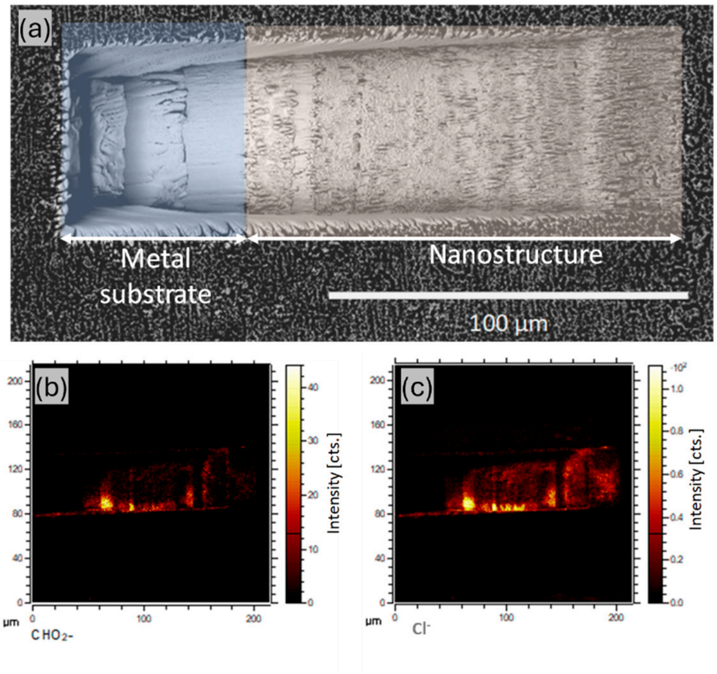

Figure S1: Back scattered electron (BSE) image of the top view on the milled material created by FIB, indicating areas of nanostructure and metal substrate; b,c) ToF – SIMS images showing distribution within the milled area of CHO_2^- fragment (b) and Cl^- fragment (c) as indicator for the presence of DCF throughout the structure. The colour scale corresponds to the fragment signal intensity

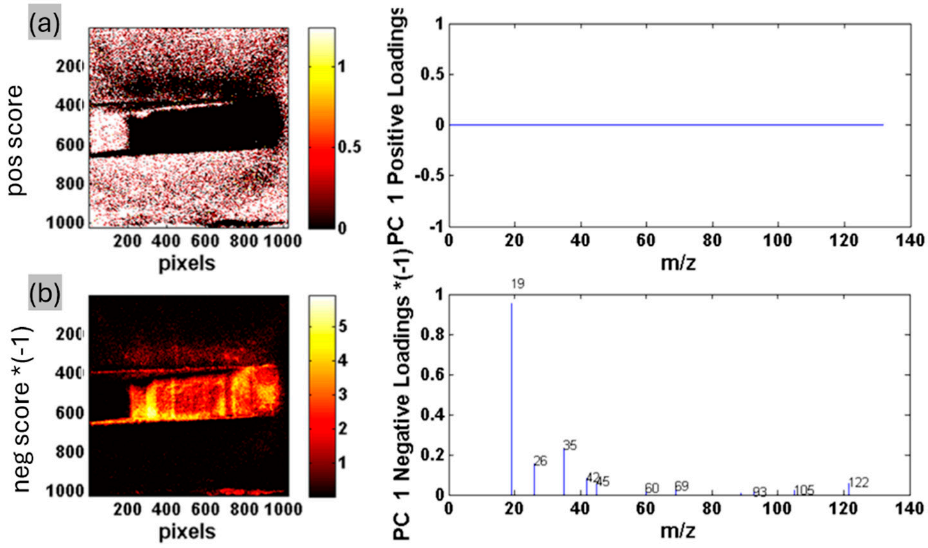

Figure S2: ToF – SIMS, PCA of inclined slope of FIB-cut. PC1 contains topography information solely (77% variance captured by PC1; (a) top surface, (b) all signals from FIB-crater).
